# Lipid and hemolysis parameters predicting acute chest syndrome in adulthood with sickle cell disease

**DOI:** 10.1186/s12944-024-02135-8

**Published:** 2024-05-16

**Authors:** Guillaume Feugray, Maximilien Grall, Cécile Dumesnil, Valéry Brunel, Ygal Benhamou, Muriel Quillard Muraine, Paul Billoir

**Affiliations:** 1 Department of General Biochemistry, Normandie Univ, UNIROUEN, INSERM U1096 EnVI, CHU Rouen, Rouen, F-76000 France; 2 Department of Internal Medicine, CHU Rouen, Rouen, F-76000 France; 3 Department of Pediatric Onco-Hematology, CHU Rouen, Rouen, F-76000 France; 4 Department of General Biochemistry, CHU Rouen, Rouen, F-76000 France; 5 Department of Internal Medicine, Normandie Univ, UNIROUEN, INSERM U1096, CHU Rouen, Rouen, F-76000 France; 6 Department of General Biochemistry, Normandie Univ, UNIROUEN, INSERM U1404 INSERMU1073 ADEN, CHU Rouen, CIC-CRB, Rouen, F-76000 France; 7 Normandie Univ, UNIROUEN, INSERM U1096 EnVI, CHU Rouen, Vascular Hemostasis Unit, Rouen, F-76000 France; 8 Service de Biochimie, Centre hospitalier Universitaire Charles Nicolle, 1 rue de Germont, Rouen, 76031 France

**Keywords:** Sickle cell disease, Acute chest syndrome, Lipid parameters, Inflammatory, Hemolysis

## Abstract

Sickle cell disease (SCD) is a lifelong blood disorder affecting approximately 100,000 people in the United States and is one of the most common monogenic diseases. A serious complication of SCD is acute chest syndrome (ACS). ACS is a condition with a high rate of morbidity and mortality. The aim of the study was to assess hemolysis and lipid parameters in a cohort of confirmed SCD patients to predict ACS development in the following year.

Standard lipid were performed (triglycerides, total cholesterol, high-density cholesterol, low-density cholesterol) panel to calculate of non-HDL-C, large buoyant LDL cholesterol (lbLDL-C) and small dense LDL cholesterol (sdLDL-C) with Sampson equation. Hemolysis and hematologic parameters were also evaluated.

Among 91 patients included between September 2018 and June 2021, thirty-seven patients had history of ACS and 6 patients developed ACS during following year. In unadjusted logistic regression, total bilirubin was associated with ACS occurrence (RR: 1.2 [1.05–1.51] *p* = 0.013). Concerning lipid profile, non-HDL-C (RR: 0.87 [0.0.67–0.99] *p* = 0.04) and sdLDL-C (RR: 0.78 [0.49–0.96] *p* = 0.03) were associated with ACS occurrence decrease. C-reactive protein was associated with ACS occurrence (RR: 1.27 [1.065–1.85] *p* = 0.011).

Based on these findings, this study demonstrated that several biomarker easily available can be used at steady state to predict ACS in the following year. The validation of these results are required to ensure the reproducibility of the findings.

## Introduction

Sickle cell disease (SCD) is a lifelong blood disorder affecting approximately 100,000 people in the United States and is one of the most common monogenic diseases [1]. SCD is a genetic disorder characterized by the production of abnormal hemoglobin S (HbS), leading to the formation of sickle red blood cells (RBCs). The disease is defined by HbS which has reduced solubility when deoxygenated compared to normal hemoglobin A, inducing organ ischemia [2]. Factors promoting erythrocyte sickling include hypoxemia, dehydration, inflammation, infection and fever [3, 4].

A major complication of SCD is acute chest syndrome (ACS). ACS is a form of acute lung injury that encompasses vaso-occlusive events within the pulmonary vasculature [5]. Approximately 50% of patients with SCD will have an episode of ACS during their lifetime [6]. ACS is a serious condition with a high rate of morbidity and mortality [7]. Incidence of ACS is lower in adults compared to children but mortality associated to these episodes is higher in adults [8]. The interest to study in each population is important to distinguish particularity of each one. Several biomarkers are associated with SCD complications [9]. However, few laboratory biomarkers are evaluated for ACS prediction. Most of these biomarkers have only been assessed on arrival at the emergency department or during a vaso-occlusive crisis (VOC) [10–14].

While the pathophysiology of SCD primarily involves the polymerization of hemoglobin, emerging evidence suggests that lipid metabolism alterations play a crucial role in the disease progression [15, 16]. Lipid metabolism abnormalities in SCD are believed to result from a combination of factors, including genotype, chronic hemolysis, inflammation, oxidative stress and altered cellular membrane composition [17–20]. These factors collectively contribute to dysregulated lipid homeostasis, leading to imbalances in lipid profiles and potentially influencing disease severity and complications [15, 21–24]. One crucial aspect of lipid abnormalities in SCD is the disruption of membrane phospholipids. RBCs during SCD exhibit higher levels of phosphatidylserine due to altering membrane stability and impairing the integrity of these cells [16, 25]. In addition to altered lipid profiles, studies reported associations between circulating lipid abnormalities and clinical manifestations in SCD. High levels of triglycerides (TG) is correlated to pulmonary hypertension and endothelial dysfunction [15]. Furthermore, dyslipidemia in SCD has been associated with reduced nitric oxide bioavailability, oxidative stress, inflammation impaired vasodilation, which can contribute to the development of complications, such as pneumonia, leg ulcers and vasculopathy [9, 24]. In addition, inflammation contributes to ACS development. It is reported that interleukine-6 (IL-6) in blood and sputum is predictor of ACS development [10, 26]. In preliminary study, Styles et al. demonstrated the interest of soluble phospholipase A2 (PLA_2_) in the prediction of ACS for 21 admissions for VOC of which 6 have developed an ACS, suggesting a role of lipid mediators in this complication [27]. However, the main limitation is that PLA_2_ measurement is not widely available in all hospital laboratories.

The aim of the study was to assess hemolysis and lipid parameters at steady state as a predictive biomarker could be used in ACS development in the following year.

## Methods

### Study design and patients

**Fig. 1:**
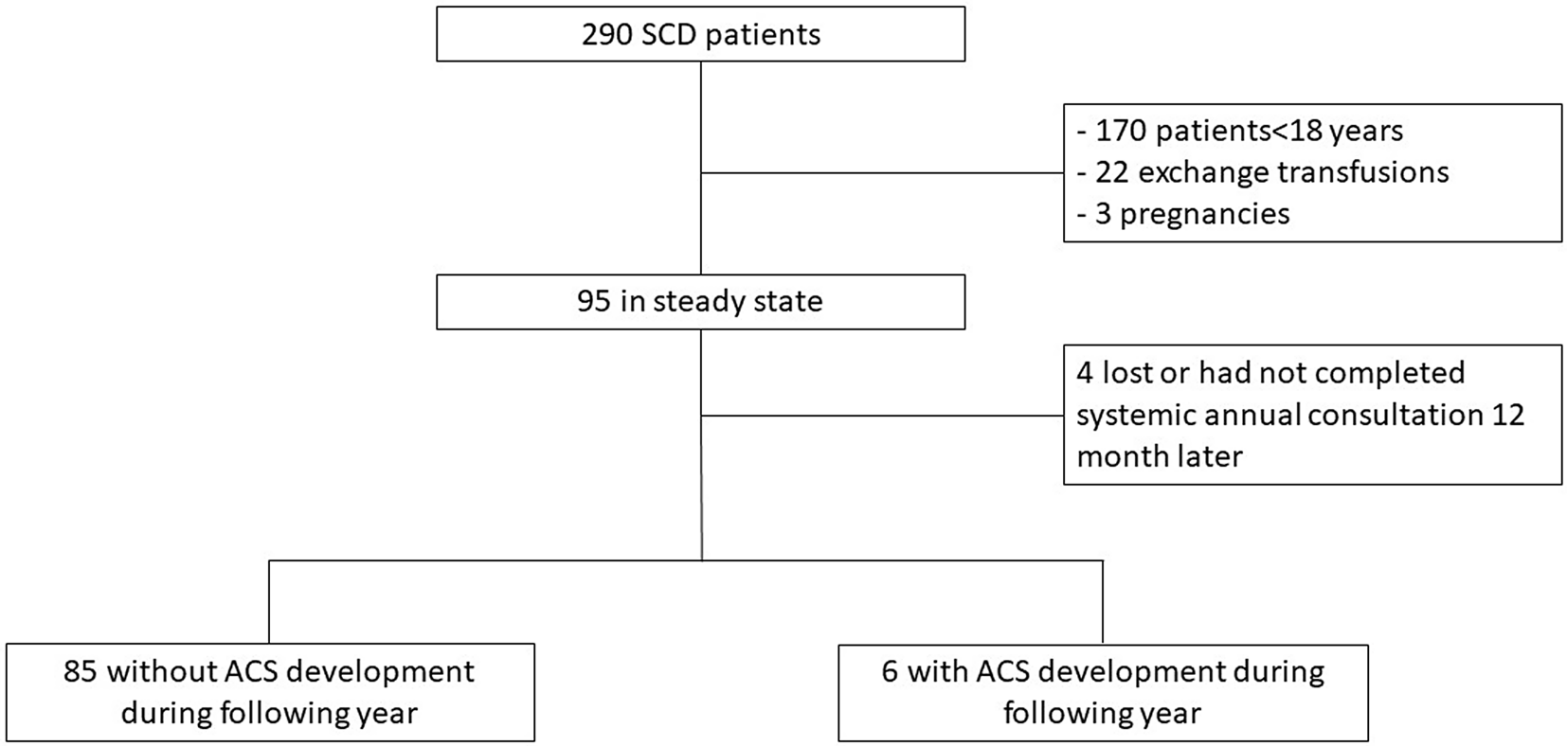
Flowchart of study design

Patients were treated for SCD at Rouen University Hospital between September 2018 and June 2021. Patients were included at steady state in our university hospital’s Sickle-Cell Referral Center. All patients received a systematic annual consultation for global injury evaluation and in which ACS events are reported. We included 290 SCD patients. Patient treated by exchange transfusion (*n* = 22), pregnant women (*n* = 3) and patients < 18 years (*n* = 170) were excluded. Any patients did not reported diabetes or familial hypercholesterolemia. Among 95 patients, four patients are lost or had not completed systemic annual consultation 12 months later. Patients with or without ACS development in the following year are separated (Fig. 1).

Clinical information included age, sex, SCD phenotype, past medical history including sickle cell complications and laboratory results at initial visit. Follow-up was strictly observational and a minimum of 12 months of follow-up was required to be included. Acute chest syndrome informations were collected in systemic annual consultation or in medical records.

Diagnosis of ACS includes a new segmental radiodensity on chest imaging and one of the following [8]:

1) fever (38.5 °C);

2) hypoxemia (> 2% decrease in SpO2 or PaO2 < 60 mmHg);

3) tachypnea;

4) cough, chest pain, rales, wheezes.

The study was performed in accordance with the Declaration of Helsinki. The institutional review board approved the study (Authorization protocol number: E2021-78) and registered on ClinicalTrials.gov (NCT05376046).

### Samples and analysis

Dipotassium EDTA tubes (BD Vacutainer CAT, Plymouth) were used for blood counts. Clot activator tubes (BD Vacutainer CAT, Plymouth) were used for lipid parameters and lithium heparin tubes (BD Vacutainer LH, Plymouth) for biochemistry. Analysis were performed the day of collection.

### Lipid measurement and estimation

Subjects were withdrawn after overnight fasting. Clot activator tubes were centrifuged at 1700 g, 4 °C, 10 min. Triglycerides (TG), total cholesterol (TC) and high-density lipoprotein cholesterol (HDL-C) were performed enzymatically on the Cobas® 8000 chemistry analyzer (Roche Diagnostics, Mannheim, Germany). Low-density lipoprotein cholesterol (LDL-C) was calculated by the Friedewald equation. Additionaly, we estimated the others parameters using following formulas:

$$Non - HDL - C{\text{ }} = {\text{ }}TC{\text{ }} - {\text{ }}HDL - C$$

$$\eqalign{ Atherogenic{\text{ }}Index{\text{ }}of{\text{ }}Plasma{\text{ }}\left( {AIP} \right){\text{ }} = & \cr & {\text{ }}Log{\text{ }}\left( {TG/HDL - C} \right) \cr}$$

$$\eqalign{ Triglyceride{\text{ }}Rich{\text{ }}Lipoprotein{\text{ }}\left( {TRL - C} \right){\text{ }} & \cr= {\text{ }}Non - HDL - C{\text{ }}-{\text{ }}LDL - C \cr}$$

Standard lipid panel was used to calculate of large buoyant LDL cholesterol (lbLDL-C) and small dense LDL cholesterol (sdLDL-C) with Sampson Eqs. [28, 29] on the following website: https://figshare.com/articles/software/Sampson_sdLDLC_Equation_Calculator_xlsx/12888293.

### Laboratory parameters

We assessed liver functions with alanine aminotransferase (ALT), aspartate aminotransferase (AST), alkaline phosphate (ALP) and gamma-glutamyl transferase (GGT). Inflammation and hemolysis was evaluated using plasma C-reactive protein (CRP), ferritin, lactate dehydrogenase activity (LDH), haptoglobin, total (BT) and direct bilirubin (BD) levels on Cobas® 8000 chemistry analyzer (Roche Diagnostics, Mannheim, Germany). Indirect bilirubin (BI) was calculated using the following formula:

$$\eqalign{ indirect{\text{ }}bilirubin{\text{ }} & = {\text{ }}total{\text{ }}bilirubin \cr & \quad -{\text{ }}direct{\text{ }}bilirubin \cr}$$

Hematology parameters were performed on XN-9000 (Sysmex®, Villepinte, France)

### Statistical analysis

The Shapiro–Wilk test was used to test normality of continuous variables. Normally distributed data were presented as mean ± standard deviations (SDs) and non-Gaussian variables as median ± interquartile range (IQR). We performed analysis to test relationships between biomarkers and ACS. Unadjusted subgroup comparisons were analysed using the Mann–Whitney U test. Unadjusted logistic regression analysis of clinical outcome (ACS) was performed using the following variables as predictors: lipid profile and hemolysis parameters. Receiver operating characteristic (ROC) curves were built for significant clinical characteristics. Data were analyzed using Graphpad Prism 10.0.2.

## Results

### Demographic and clinical characteristics

A total of 91 patients at steady state with SCD were included in this study. The main clinical and biological characteristics are shown in Table 1. The median age was 34.0 ± 11.81 years. A total of 48.35% of the cohort was male. Among the 91 patients at steady state, 35 were S/S or S/β^0^, 24 were S/S with α^3.7^ co-inheritance and 32 were S/C or S/β^+^. Fifty-one (56.04%) were treated by hydroxyurea. Past history of osteonecrosis (32.96%), retinopathy (31.86%), vasculopathy (17.58%), cholecystectomy (45.05%), splenectomy (3.29%) and ACS (40.65%) were reported.

**Table 1:** Characteristic of study population

	*n* = 91
**Clinical characteristics**	
Age (years)	34.0 ± 11.81
Male n (%)	44 (48.35)
Hydroxyurea n (%)	51 (56.04)
Osteonecrosis n (%)	30 (32.96)
Retinopathy n (%)	29 (31.86)
Vasculopathy n (%)	16 (17.58)
Cholecystectomy n (%)	41 (45.05)
Splenectomy n (%)	3 (3.29)
ACS n (%)	37 (40.65)
**Genotype**	
S/S-Sβ^0^	35 (38.46)
S/S with α^3.7^ deletion	24 (26.37)
S/C-S/β^+^	32 (35.16)
**Blood counts**	
RBC (10^12^/L)	3.23 [2.70–4.26]
Hemoglobin (g/dL)	9.5 [8.4–10.8]
Hematocrit (%)	28 [24–32]
Platelets (10^9^/L)	303 [200–380]
Leukocytes (10^9^/L)	7.3 [5.8–9.4]
Neutrophils (10^9^/L)	3.9 [2.8–5.1]
Lymphocytes (10^9^/L)	2.6 [1.8–3.2]
Monocytes (10^9^/L)	0.7 [0.5-1.0]
Reticulocytes (10^9^/L)	308 [198–539]
Immature reticulocyte fraction (10^9^/L)	65.6 [35.9-127.3]
**Biochemistry parameters**	
Total bilirubin (µmol/L)	24.0 [14.5–44.0]
Direct bilirubin (µmol/L)	11.0 [8.0-12.5]
Indirect bilirubin (µmol/L)	26.5 [16.5–39.8]
LDH (U/L)	333 [212–436]
C-reactive protein (mg/L)	4.5 [2.0–7.0]
Ferritin (µg/L)	114 [52–249]
ASAT (U/L)	37 [16, 26–48]
ALAT (U/L)	23 [16–30]
γGT (U/L)	36 [24–77]
ALP (U/L)	74 [58–101]
Urea (mmol/L)	3.2 [2.4–4.2]
Creatinine (µmol/L)	59.0 [49.5–72.5]
HbS (%)	69.0 [47.7–87.6]
**Lipid profile**	
TG (mg/dL)	90.0 [69.0-119.0]
TC (mg/dL)	120.0 [107.5–150.0]
HDL-C (mg/dL)	44.0 [36.8–52.0]
Estimated LDL-C (mg/dL)	61.0 [46.0-75.8]
TC/HDL-C	2.8 [2.4–3.7]
LDL-C/HDL-C	1.36 [0.96–1.99]
Non-HDL-C (mg/dL)	79.0 [63.0-96.5]
AIP	0.31 [0.17–0.51]
TRL-C (mg/dL)	18.5 [13.8–25.0]
Estimated lbLDL-C (mg/dL)	41.20 [26.48–55.40]
Estimated sdLDL-C (mg/dL)	20.80 [17.15–24.48]

Data are expressed as median ± [IQR] except for age (mean ± SD) and clinical characteristics, n is the total number of patients (%), ACS: acute chest syndrome, RBC: red blood cells, LDH: lactate deshydrogenase, ALAT: alanine amino-transferase, ASAT: aspartate amino-transferase, γGT: gamma glutamyl-transferase, HbS: hemoglobin S, TG: triglyceride, TC: total cholesterol, AIP: Atherogenic Index of Plasma, TRL-C: Triglyceride Rich Lipoprotein

### Factors associated with past history of acute chest syndrome

Differences between patients with history of ACS (*n* = 37) and without (*n* = 54) were compared (Table 2). Past history of cholecystectomy (*p* = 0.003) and patients S/S-S/β^0^ genotype (*p* = 0.049) reported higher history of ACS as expected. Consistently, lower ACS history was reported in patients S/C-S/β^+^ subgroup (*p* < 0.0001).

**Table 2:** Past of acute chest syndrome

	Without history of ACS *n* = 54	With history of ACS *n* = 37	*P* value
**Clinical characteristics**			
Age (years)	32.5 ± 13.8	36.0 ± 8.2	0.13
Male n (%)	24 (44.4)	20 (54.0)	0.39
Hydroxyurea n (%)	25 (46.2)	26 (70.2)	**0.03**
Osteonecrosis n (%)	15 (27.7)	15 (40.5)	0.26
Retinopathy n (%)	19 (35.1)	10 (27.0)	0.49
Vasculopathy n (%)	8 (14.8)	8 (21.6)	0.42
Cholecystectomy n (%)	17 (31.4)	24 (64.8)	**0.003**
Splenectomy n (%)	2 (3.7)	1 (2.7)	1
**Genotype**			
S/S-S/β^0^	16 (29.6)	19 (51.3)	**0.049**
S/S with α^3.7^ deletion	10 (18.5)	14 (37.8)	0.053
S/C-S/β^+^	28 (51.8)	4 (10.8)	**< 0.0001**
**Blood counts**			
RBC (10^12^/L)	3.84 [2.94–4.41]	2.95 [2.56–3.72]	**0.0045**
Hemoglobin (g/dL)	10.3 [8.6–11.7]	9.2 [7.7–10.1]	**0.002**
Hematocrit (%)	30 [25–32]	27 [23–30]	**0.01**
Platelets (10^9^/L)	284 [197–345]	338 [209–450]	0.051
Leukocytes (10^9^/L)	6.8 [5.4–8.2]	9.3 [7.1–11.4]	**0.0001**
Neutrophils (10^9^/L)	3.4 [2.6–4.3]	4.7 [3.5–6.4]	**0.0001**
Lymphocytes (10^9^/L)	2.3 [1.5–2.9]	2.7 [2.1–3.8]	**0.013**
Monocytes (10^9^/L)	0.7 [0.4–0.9]	0.9 [0.6–1.2]	**0.0077**
Reticulocytes (10^9^/L)	151 [97–258]	308 [179–369]	**< 0.0001**
Immature reticulocyte fraction (10^9^/L)	42.9 [29.1–95.1]	129.5 [58.6-149.4]	**< 0.0001**
**Biochemistry parameters**			
Total bilirubin (µmol/L)	21.0 [12.0–35.0]	37.0 [22.3–52.5]	**0.0003**
Direct bilirubin (µmol/L)	10.0 [8.0–12.0]	11.0 [8.0-13.3]	0.27
Indirect bilirubin (µmol/L)	24.0 [16.0-33.8]	31 [17.3–57.0]	0.21
LDH (U/L)	271 [205–375]	416 [328–559]	**< 0.0001**
C-reactive protein (mg/L)	3.0 [1.0–6.0]	6.5 [2.8–10.3]	**0.006**
Ferritin (µg/L)	97 [43–204]	156 [84–351]	0.06
ASAT (U/L)	29 [16, 23–37]	43 [34–58]	**< 0.0001**
ALAT (U/L)	22 [15–29]	26 [17–33]	0.14
γGT (U/L)	27 [16, 21–48]	58 [32–89]	**0.01**
ALP (U/L)	67 [54–88]	94 [70–110]	**0.0001**
Urea (mmol/L)	3.3 [2.7–4.5]	2.8 [2.3–4.2]	0.14
Creatinine (µmol/L)	59.0 [51.5–78.0]	59.0 [48.0–71.0]	0.45
HbS (%)	49.1 [46.8–84.1]	85.1 [72.2–92.5]	**0.0017**
**Lipid profile**			
TG (mg/dL)	80.0 [63.0-110.5]	102.0 [80.0-127.0]	**0.031**
TC (mg/dL)	135.0 [110.0-150.0]	110.0 [100.0-130.0]	**0.0007**
HDL-C (mg/dL)	46.5 [38.0-55.3]	39.0 [31.5–46.0]	**0.0074**
Estimated LDL-C (mg/dL)	65.5 [49.0-87.3]	55.0 [36.3–66.8]	**0.0077**
TC/HDL-C	2.8 [2.4–3.7]	2.8 [2.4–3.7]	0.73
LDL-C/HDL-C	1.3 [1.0–2.0]	1.5 [0.8–2.1]	0.55
Non-HDL-C (mg/dL)	84.5 [64.0-104.3]	73.0 [57.0-88.5]	**0.027**
AIP	0.23 [0.09–0.48]	0.41 [0.23–0.52]	**0.0065**
TRL-C (mg/dL)	18.0 [12.8–23.3]	19.5 [15.5–25.8]	0.23
Estimated lbLDL-C (mg/dL)	47.05 [29.90-62.25]	35.05 [19.95–44.33]	**0.0017**
Estimated sdLDL-C (mg/dL)	21.0 [18.0-26.5]	20.6 [16.1–24.2]	0.24

Data are expressed as median ± [IQR] except for age (mean ± SD) and clinical characteristics, n is the total number of patients (%), ACS: acute chest syndrome, RBC: red blood cells, LDH: lactate deshydrogenase, ALAT: alanine amino-transferase, ASAT: aspartate amino-transferase, γGT: gamma glutamyl-transferase, HbS: hemoglobin S, TG: triglyceride, TC: total cholesterol, AIP: Atherogenic Index of Plasma, TRL-C: Triglyceride Rich Lipoprotein

History of ACS had significant higher leukocytes (*p* = 0.0001), neutrophils (*p* = 0.0001), lymphocytes (*p* = 0.013), monocytes (*p* = 0.0077), total bilirubin (*p* = 0.0003), LDH (*p* < 0.0001), CRP (*p* = 0.006), HbS (*p* = 0.0017), TG (*p* = 0.031) and API (*p* = 0.0065). Likewise, history of ACS is associated with a significant lower RBC (*p* = 0.0045), hemoglobin (*p* = 0.002), hematocrit (*p* = 0.01), TC (*p* = 0.0007), HDL-C (*p* = 0.0074), LDL-c (*p* = 0.0077), non-HDL-C (*p* = 0.027) and estimated lbLDL-C (*p* = 0.0017).

### Biomarkers as predictors of acute chest syndrome occurrence

We evaluated biomarkers to predict ACS (Table 3). Six patients developed ACS during following year. On laboratory results, we found that patients who developed ACS in the following year had significant higher total bilirubin (*p* = 0.014), LDH (*p* = 0.027) and C-reactive protein (*p* = 0.034). Likewise, we found that patients who develop ACS in the following year presented significant lower non-HDL-C (*p* = 0.043).

**Table 3:** Laboratory parameters associated with acute chest syndrome development in the following year

	Without ACS in the following year *n* = 85	With ACS in the following year *n* = 6	*P* value
**Clinical characteristics**			
Age (years)	34.0 ± 11.8	33.0 ± 13.6	0.93
Male n (%)	41 (48.2)	3 (50.0)	1
Hydroxyurea n (%)	47 (55.3)	4 (66.6)	0.69
Osteonecrosis n (%)	26 (30.6)	4 (66.6)	0.089
Retinopathy n (%)	29 (34.1)	0 (0)	0.17
ACS n (%)	34 (40.0)	3 (50.0)	0.68
Vasculopathy n (%)	15 (17.6)	1 (16.6)	1
Cholecystectomy n (%)	39 (45.9)	2 (33.3)	0.68
Splenectomy n (%)	3 (3.5)	0 (0)	1
**Genotype**			
S/S-S/β^0^	31 (36.5)	4 (66.6)	0.19
S/S with α^3.7^ deletion	23 (27.0)	1 (16.6)	1
S/C-S/β^+^	31 (36.5)	1 (16.6)	0.41
**Blood counts**			
RBC (10^12^/L)	3.24 [2.82–4.35]	2.69 [2.61–3.33]	0.11
Hemoglobin (g/dL)	9.7 [8.5–10.8]	9.1 [7.6–10.4]	0.43
Hematocrit (%)	28.0 [24.5–32.0]	26.5 [22.0–29.0]	0.28
Platelets (10^9^/L)	304 [204–383]	230 [146–370]	0.36
Leukocytes (10^9^/L)	7.3 [5.7–9.4]	8.1 [6.8–10.6]	0.39
Neutrophils (10^9^/L)	3.9 [2.7–5.1]	4.8 [3.8-7.0]	0.08
Lymphocytes (10^9^/L)	2.6 [1.8–3.2]	2.0 [1.8–3.3]	0.68
Monocytes (10^9^/L)	0.7 [0.5-1.0]	0.9 [0.5-1.0]	0.85
Reticulocytes (10^9^/L)	179 [115–307]	301 [255–394]	0.07
Immature reticulocyte fraction (10^9^/L)	59.2 [34.5-125.3]	127.0 [93.6-184.1]	**0.02**
**Biochemistry parameters**			
Total bilirubin (µmol/L)	23.0 [14.0–44.0]	48.0 [37.75–123.5]	**0.014**
Direct bilirubin (µmol/L)	10.0 [8.0–12.0]	13.5 [7.5-39.75]	0.28
Indirect bilirubin (µmol/L)	26.5 [15.75–38.25]	34.5 [23.0–57.0]	0.23
LDH (U/L)	318 [209–417]	487 [415–525]	**0.027**
C-reactive protein (mg/L)	4.0 [2.0–7.0]	9.0 [5.5–16.5]	**0.034**
Ferritin (µg/L)	113 [49–245]	407 [100–673]	0.17
ASAT (U/L)	34 [16, 26–49]	43 [38–48]	0.19
ALAT (U/L)	22 [16–30]	28 [21–30]	0.49
γGT (U/L)	36 [24–75]	71 [41–119]	0.16
ALP (U/L)	73 [58–101]	94 [70–213]	0.15
Urea (mmol/L)	3.2 [2.4–4.1]	3.6 [2.6–5.6]	0.48
Creatinine (µmol/L)	59.0 [49.0–71.0]	65.0 [52.0-94.8]	0.49
HbS (%)	68.6 [47.5–87.1]	81.0 [78.1–93.6]	0.25
**Lipid profile**			
TG (mg/dL)	95.0 [69.0-121.0]	87.0 [77.0-93.3]	0.46
TC (mg/dL)	125.0 [110.0-150.0]	110.0 [87.5-122.5]	0.06
HDL-C (mg/dL)	43.5 [36.3–51.8]	48.0 [35.3–57.0]	0.60
Estimated LDL-C (mg/dL)	61.0 [46.0–81.0]	46.0 [22.5–64.8]	0.07
TC/HDL-C	2.8 [2.4–3.7]	2.2 [1.9–3.2]	0.11
LDL-C/HDL-C	1.4 [1.0–2.0]	0.9 [0.6–1.7]	0.14
Non-HDL-C (mg/dL)	79.0 [63.3–98.8]	62.0 [39.0-83.3]	**0.043**
AIP	0.32 [0.15–0.51]	0.26 [0.14–0.39]	0.52
TRL-C (mg/dL)	18.5 [13.3–25.0]	18.0 [14.0–21.0]	0.61
Estimated lbLDL-C (mg/dL)	41.20 [26.63–55.85]	31.50 [8.18–44.15]	0.09
Estimated sdLDL-C (mg/dL)	20.90 [17.40-25.38]	16.25 [13.03–21.73]	0.06

Data are expressed as median ± [IQR] except for age (mean ± SD) and clinical characteristics, n is the total number of patients (%), ACS: acute chest syndrome, RBC: red blood cells, LDH: lactate deshydrogenase, ALAT: alanine amino-transferase, ASAT: aspartate amino-transferase, γGT: gamma glutamyl-transferase, HbS: hemoglobin S, TG: triglyceride, TC: total cholesterol, AIP: Atherogenic Index of Plasma, TRL-C: Triglyceride Rich Lipoprotein

**Fig. 2:**
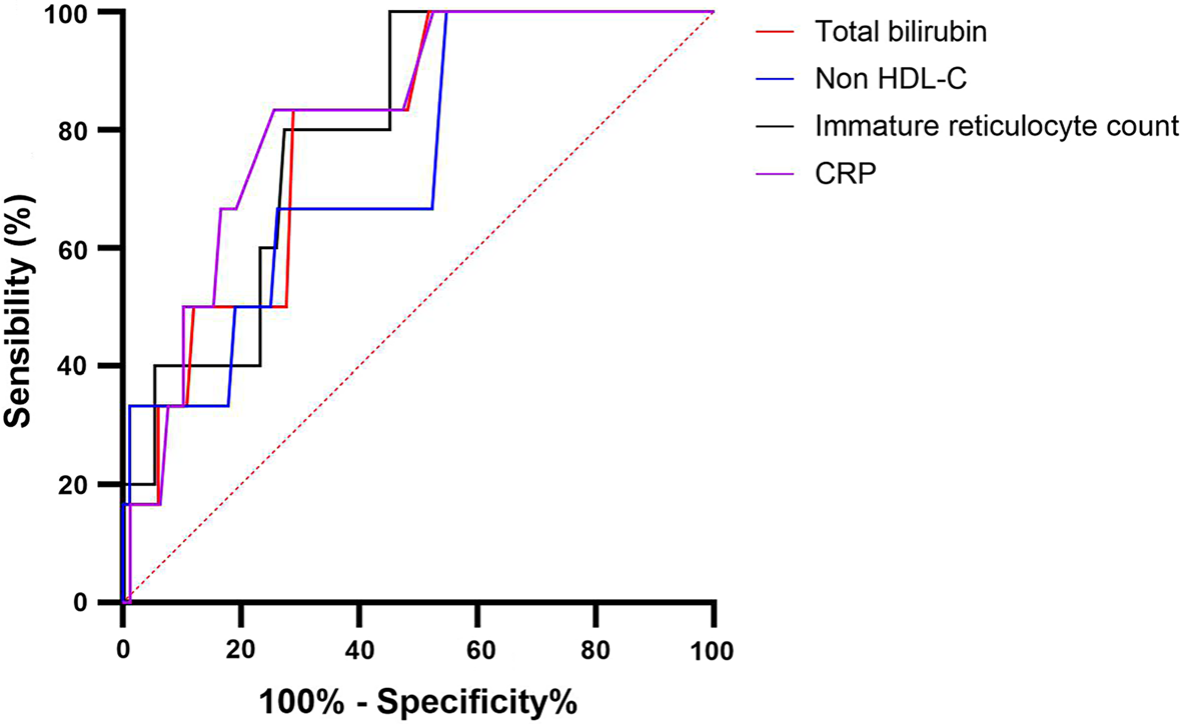
ROC curve of acute chest syndrome prediction by total bilirubin, immature reticulocyte fraction, non-HDL-C and C-reactive protein (CRP). Comparison with no ACS development and ACS development in the following year

**Fig. 3:**
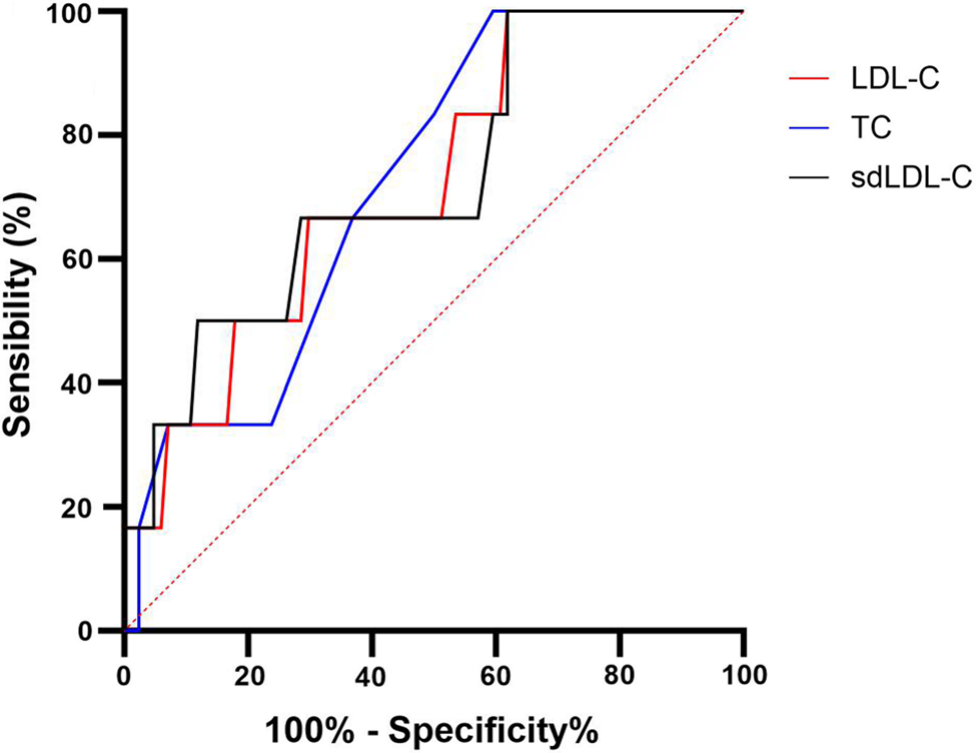
ROC curve of acute chest syndrome prediction LDL-C, total cholesterol (TC) and sdLDL-C. Comparison with no ACS development and ACS development in the following year

In unadjusted logistic regression, total bilirubin was associated with ACS occurrence (RR: 1.2 [1.05–1.51] *p* = 0.013). Concerning lipid parameters, non-HDL-C (RR: 0.87 [0.67–0.99] *p* = 0.04) and sdLDL-C (RR: 0.78 [0.49–0.96] *p* = 0.03) protected from ACS occurrence. C-reactive protein was associated with ACS occurrence (RR: 1.27 [1.065–1.85] *p* = 0.011). No others parameters were associated with ACS occurrence. The prediction of ACS was determined with a ROC curve (Fig. 2): a total bilirubin > 39 mg/L (AUC: 0.79, sensitivity: 83.3%, specificity: 71.1%), non-HDL-C < 64.5 mg/dL (AUC: 0.746, sensitivity: 66.7%, specificity: 73.8%), CRP > 8.5 mg/L (AUC: 0.82, sensitivity: 66.7%, specificity: 83.3%) and an immature reticulocyte count of > 117.5 10^9^/L (AUC: 0.80, sensitivity: 80%, specificity: 72.6%) were associated with ACS. Additionally, significant ROC curves for lipid parameters were reported with sdLDL-C < 14.85 mg/dL (AUC: 0.73, sensitivity: 50%, specificity: 88.1%), LDL-C < 44 mg/dL (AUC: 0.72, sensitivity: 50%, specificity: 82.1%) and TC < 115 mg/dL (AUC: 0.72, sensitivity: 66.7%, specificity: 83.3%) were associated with ACS (Fig. 3).

## Discussion

The aim of the study was to assess lipid profile in SCD patients at steady state to investigate whether a predictive biomarker could be used to predict the prediction of ACS development. The association of standard and calculated lipid profile at steady state showed an interesting correlation with ACS development in the following year. Interestingly, a significant lower non-HDL-C in patients who developed ACS in the following year was reported. Moreover, TC, LDL-C, non-HDL-C and sdLDL-C are protected from ACS occurrence with interesting ROC curve results. These results suggest the implication of dyslipidemia in ACS occurrence.

Vaso-occlusive crisis is the leading cause of hospitalization in patients with SCD and nearly 15% of these patients develop ACS within the 3 days of admission [2]. Overall, ACS accounts for approximately 25% of deaths in patients with SCD [30]. The incidence of ACS is higher in children aged before 4 years compared to adults, with three times more events per years [7, 31]. However, severity and mortality rate are much higher in adults with SCD and ACS compared to children. A possible complication is multiorgan failure, marked by acute dysfunction of at least two organ systems, principally acute kidney injury and hepatic dysfunction [32].

Lipid parameters are classically used to assess cardiovascular risk. However, it can be a diagnostic or prognostic marker for others diseases [33, 34]. It is reported that more frequent positive history of ACS was observed in SCD patients with TG level higher than 1.50 g/L [35]. Here, patients with history past of ACS presented lower hemoglobin, TC, HDL-C, LDL-C, non-HDL-C and higher leukocytes, neutrophils, reticulocytes, total bilirubin, LDH, TG and AIP.

Kuypers described that RBC membrane is complex mixture of lipids and proteins more particularly phospholipids. Alteration of lipid bilayer in hemoglobinopathies lead to apoptosis [16]. This alteration induces to anemia and increase exposure of phosphatidyl-serine leading to vascular dysfunction. Reduction of plasma cholesterol in SCD is associated with an increase of cholesterol in RBC membrane [36]. As reported by Westerman, hypocholesterolemia is associated with anemia but equally with hematocrit, both hemolytic and hypoproliferative [37]. Moreover, a reduction of lecithin cholesterol acyl-transferase (LCAT) activity is associated with deformability. Incubation of RBC from SCD patients with HDL-C improves their deformability and reduces reactive oxygen species [36]. HDL regulates the cholesterol/phospholipid ratio removing excess membrane cholesterol from RBC. Vendrame et al. demonstrated that SCD patients exhibit higher oxidative-LDL compared to health controls [38]. Additionally, LDL fraction contained higher concentrations of heme unlike HDL which contained more hemopexin suggesting an important role of HDL fraction in the defense against heme induced endothelial dysfunction [38]. To complete the lipid homeostasis in the pathophysiology, oxysterols, oxidized derivates of cholesterol, affect cholesterol metabolism and eryptosis. Indeed, oxysterols is inversely correlated with hemoglobin and correlated with LDH [39].

Few biomarkers were associated with ACS prediction. Castro et al. reported in 3751 patients in prospective multicenter study that leukocytes count at steady state is associated with ACS [40]. In this study, we found that patients who develop ACS in the following year reported significant higher CRP. Our ROC curve presented interesting performance with CRP measurement (sensitivity: 66.7%, specificity: 83.3%) reinforcing the inflammatory hypothesis.

Previously, we reported an algorithm based on LDH and hemolysis index at steady state to predict VOC [41]. Patients who develop ACS in the following year reported significant higher total bilirubin and LDH. Previous studies demonstrated the interest of reticulocyte and coagulation parameters in prediction of VOC [42, 43]. Unadjusted logistic regression confirmed that total bilirubin was associated with ACS occurrence. The ROC curve presented interesting performances for total bilirubin (sensitivity: 83.3%, specificity: 71.1%) and immature reticulocyte count of > 117.5 10^9^/L (sensitivity: 80%, specificity: 72.6%).

In addition, lipid profile is implicated in others SCD complications notably pulmonary hypertension [15, 44]. Moreover, serum amyloid A / apolipoprotein A1 ratio increased in 81% of patients with SCD during acute painful episodes [45]. It is reported that TG/HDL-C ratio is correlated with LDH, leukocytes and blood flow velocity [46]. Moreover, ACS and VOC were more frequent in SCD patients exhibiting higher TG/HDL-C values [46]. Thus, TG/HDL-C ratio is a potential biomarker of severity of disease [46]. Here an association between lipid profile and ACS prediction was demonstrated with ROC curves for non-HDL-C (sensitivity: 66.7%, specificity: 73.8%), sdLDL-C (sensitivity: 50%, specificity: 88.1%), LDL-C (sensitivity: 50%, specificity: 82.1%) and TC < 115 mg/dL (sensitivity: 66.7%, specificity: 83.3%) were associated with ACS. More recently, Olabode et al. reported that high pulse pressure is correlated with TG/HDL-C ratio in sickle cell trait and SCD [47]. Authors conclude that TG/HDL-C ratio can be a salient risk factor that promote arterial stiffness.

The immediate aim of treatment in ACS is to prevent or reverse acute respiratory failure. The current standard of care for ACS used in hospital settings is broadly comprised of supportive care (oxygen supplementation, incentive spirometry, mechanical ventilation, hydration), transfusion therapy (simple transfusion, exchange transfusion chronic), and pharmacotherapy (analgesics, antibiotics/antivirals, corticosteroids, bronchodilators, inhaled NO) [48]. The interest to predict ACS in SCD could guide therapeutic decision-making with novel therapeutics.

### Strengths and limitations

The strengths of this study are that lipid and hemolysis parameters at steady state are easily available. The interest to predict ACS in the following year at steady state and not only at admission in emergency department as previously reported, allows for increased medical monitoring.

This study has several limitations, including a small sample size. This preliminary results need to be confirmed in larger multicenter study. However all laboratory analysis can be easily performed in most hospitals. Secondly, this study focused exclusively on ACS occurrence. Others SCD complications like pulmonary hypertension, vasculopathy and nephropathy should be interesting. ACS have several independent variables that may exert cofounding in the study notably epidemiological, clinical and treatments [49]. However, by including several genotypes, limiting as far as possible several confounding factors (i.e. pregnancy, children, diabetes) outside of a crisis (infection, VOCs) and without transfusion exchange, we tried to limit these factors.

## Conclusion

Based on these findings, this study demonstrated lipid and hemolysis parameters easily available, can be used at steady state to predict ACS in the following year. The validation of these results are required to ensure the reproducibility of the developed model.

## Data Availability

All data generated or analyzed during this study are included in this published article.
